# Ventricular Tachycardia Predicts All-Cause Mortality and Nonsudden Cardiac Death in Nonischemic Cardiomyopathy

**DOI:** 10.1016/j.jacadv.2025.102063

**Published:** 2025-08-13

**Authors:** Sharif Omara, Thomas N. Jensen, Lars Koeber, Jens J. Thune, Steen Pehrson, Bart Mertens, Usha B. Tedrow, Gerhard Hindricks, Micaela Ebert, Corrado Carbucicchio, Antonio Berruezo, Marmar Vaseghi, Kalyanam Shivkumar, Thomas Deneke, Adrianus P. Wijnmaalen, William G. Stevenson, Jens C. Nielsen, Katja Zeppenfeld

**Affiliations:** a Department of Cardiology, Leiden University Medical Centre, Leiden, the Netherlands; b Willem Einthoven Centre for Cardiac Arrhythmia Research and Management (WECAM), Leiden, The Netherlands; c Department of Cardiology, Aarhus University Hospital, Aarhus, Denmark; d Department of Clinical Medicine, Aarhus University, Aarhus, Denmark; e Cardiology, Rigshospitalet - Copenhagen University Hospital, Copenhagen, Denmark; f Department of Biomedical Data Sciences, Leiden University Medical Centre, Leiden, The Netherlands; g Cardiology, Brigham and Women's Hospital, Boston, Massachusetts, USA; h Heart Centre Leipzig, University of Leipzig, Leipzig, Germany; i Heart Centre Dresden, Faculty of Medicine and University Hospital Carl Gustav Carus, TUD Dresden University of Technology, Dresden, Germany; j Department of Clinical Electrophysiology and Cardiac Pacing, IRCCS Centro Cardiologico Monzino, Milan, Italy; k Teknon Medical Centre, Cardiovascular Institute Hospital Clinic and Heart Institute, Barcelona, Spain; l David Geffen School of Medicine at UCLA, UCLA Cardiac Arrhythmia Center, Los Angeles, California, USA; m University Heart Center Nuremberg, University Hospital of the Paracelsus Medical University, Klinikum Nuremberg, Nuremberg, Germany; n Department of Cardiology, Vanderbilt University Medical Center, Nashville, Tennessee, USA

**Keywords:** heart failure, nonischemic cardiomyopathy, nonsudden cardiovascular mortality, predictor, ventricular tachycardia

## Abstract

**Background:**

Patients with nonischemic cardiomyopathies (NICMs) are at a risk for end-stage heart failure and death from ventricular arrhythmias. Implanted defibrillators (ICDs) protect against sudden arrhythmic death, but several studies suggest that ventricular arrhythmias are associated with worse outcomes despite ICDs.

**Objectives:**

This study evaluated the relationship of ventricular tachycardia (VT) with total mortality and nonsudden cardiovascular death (NSCVD) in NICM patients with ICDs enrolled in 2 multicentre trials.

**Methods:**

Patient-level data were analyzed from 2 prospective trials: DANISH (Danish Trial to Assess the Efficacy of ICDs in Patients with Nonischemic Systolic Heart Failure on Mortality) (ICD for primary prevention of sudden death) and dilated cardiomyopathy-VT (VT ablation). Primary endpoints were all-cause mortality or heart transplantation, and the secondary endpoint was NSCVD. Analyses included the entire group with multivariable analysis, a propensity-matched subgroup with and without VT at entry, VT patients with recent-onset VT in dilated cardiomyopathy-VT, and DANISH patients experiencing VT after primary prevention ICD.

**Results:**

Among 828 patients (median age 64 years, 23.3% females, median left-ventricular ejection fraction of 36%), 148 deaths occurred during a median follow-up of 3.9 years, with 78 (53%) due to NSCVD. Despite younger age and better left-ventricular ejection fraction, VT was independently associated with a >4-fold increase in mortality or transplantation (adjusted HR: 4.27 [95% CI: 2.60-7.03]) and a 7-fold increase in NSCVD (adjusted HR: 7.28 [95% CI: 3.80-13.97]). The findings were consistent across all subgroups.

**Conclusions:**

Patients with NICM experiencing VT are at an increased risk for mortality and NSCVD, suggesting that VT can be a marker for a more severe cardiomyopathy with important clinical implications.

Patients with nonischemic cardiomyopathies (NICMs) are at a risk for progression to end-stage heart failure and sudden cardiac death (SCD) due to ventricular arrhythmias (VAs). Because implanted defibrillators (ICDs) are highly effective at terminating VAs, it seems reasonable to assume that the contribution of VAs to mortality would be reduced and that the severity of heart failure and ventricular dysfunction, rather than VA, would be the major factors associated with mortality. Several studies, however, suggest that the presence of VA is a marker for poor outcomes, but multiple confounding factors have made this assessment difficult.

With the sequential introduction of heart failure medication, SCD rates have declined significantly to an annual rate of 2.7 to 3.3/100 patient-years in patients with reduced ejection fraction.^1^, ^2^, ^3^ The proportion of sudden death relative to overall mortality is approximately 35% to 39% and stable over time, suggesting that improved heart failure treatment also reduces the risk of life-threatening arrhythmia.^1^ This reduction may be partly explained by left ventricular reverse remodeling which impacts those arrhythmic substrates that are not related to irreversible replacement fibrosis.

The recent DANISH (Danish Trial to Assess the Efficacy of ICDs in Patients with Nonischemic Systolic Heart Failure on Mortality) trial randomized patients with nonischemic heart failure, left-ventricular ejection fraction (LVEF) ≤35%, and *no prior* sustained arrhythmia to ICD or no ICD, after optimized heart failure treatment, including cardiac resynchronization therapy (CRT) in 58% of the patients. ICDs reduced SCD but failed to significantly reduce the low overall all-cause mortality, which were 4.4 and 5.0 events per 100 person-years, respectively, during long-term follow-up.^4^ In contrast, all-cause mortality of contemporary patients receiving a secondary prevention ICD *after a prior arrhythmia* appears higher, reaching 10.9% 1 year after implantation in a recent analysis.^5^ Mortality data after the first arrhythmic episode, specifically in patients with NICM, are sparse. In a recent meta-analysis of 31 observational studies including 3,055 patients with NICM referred for catheter ablation (CA) of ventricular tachycardia (VT), all-cause mortality was 8.6 per 100 person-years. Notably, these patients were younger and had a higher LVEF compared to those included in primary prevention ICD trials.^6^ Similarly, in the recent international prospective cohort study (DCM-VT) of 281 NICM patients (LVEF 36% ± 12%) followed after CA for VT, the cumulative 1-year mortality rate was 13%.

These data suggest that the occurrence of VA in patients with NICM may be an indicator of a myocardial substrate that confers an increased risk of non-SCD. We hypothesized that in patients with NICM, sustained VT is associated with a worse prognosis even after adjusting for clinical predictors of all-cause mortality.

## Methods

### Study design

Two multicentre cohorts of patients with NICM for whom all individual patient data were available were merged into one cohort: 1) patients randomized to ICD treatment in the DANISH trial, referred to as the primary prevention DCM cohort (P-DCM); and 2) patients included in the Clinical Outcomes in Patients with Dilated Cardiomyopathy and Ventricular Tachycardia study, a prospective, international, multicentre cohort study which evaluated the outcomes after VT ablation in consecutive NICM patients, referred to as the VT-DCM cohort. The results from these studies have been previously published.^4^^,^^7^ To fully use the large sample size of the 2 data sets, a patient-level analysis was performed. This allowed more detailed modeling of covariates and matching. The authors had full access to the original data sets of both studies.

The DANISH trial was an investigator-initiated, multicentre, randomized, unblinded, controlled trial that was conducted at all 5 centers in Denmark at which ICDs were implanted. The trial included symptomatic patients recruited from outpatient clinics with nonischemic systolic heart failure (LVEF ≤35%) and an increased level of N-terminal pro-brain natriuretic peptide. LVEF and N-terminal pro-brain natriuretic peptide were measured after heart failure medication was increased to target levels. Patients were included from February 2008 to June 2014. Only those patients randomized to ICD implantation were used in this analysis (n = 556).

The DCM-VT study included patients with NICM who were accepted for ablation of recurrent, symptomatic, sustained VT not controllable by medication, occurring within the 6 months before the procedure at 9 participating centers according to an intention to treat principle. Patients that were discharged after radiofrequency CA and followed in the outpatient setting were included in this analysis. There were no LVEF inclusion or exclusion criteria. The inclusion period extended from September 2013 to January 2017.

Both studies excluded patients with inflammatory, restrictive, or hypertrophic cardiomyopathies. Inclusion and exclusion criteria for each study are provided in Supplemental Table 1. The majority of patients had idiopathic NICM. Patients were to receive optimal medical therapy for heart failure and CRT consistent with guideline recommendations at the time the trials were conducted. Per definition, all VT-DCM patients had a history of VT and the P-DCM patients did not.

All patients provided informed consent for treatment according to local institutional requirements. All data were anonymized and gathered online in a secure central database. Ethical approval was obtained by the relevant scientific ethics committees for the DANISH trial (regional scientific ethics committee for the Capital Region of Denmark and the Danish Data Protection Agency) and for the DCM-VT study (Leiden Den Haag ethical committee).

### Endpoints

The primary endpoint was a composite of all-cause mortality or heart transplantation. Secondary endpoints were 1) nonsudden cardiovascular death (NSCVD) and 2) sustained VT recurrence or occurrence. In the P-DCM cohort, inclusion and follow-up started at randomization to ICD implantation or control. In the VT-DCM cohort, follow-up started after the VT ablation. Both cohorts consisted of stable patients followed in an outpatient setting.

Sustained VT was defined as the occurrence of any VT requiring therapy or recorded within the ICD monitor zone lasting >30 seconds or documented on 12-lead electrocardiography lasting >30 seconds. In the P-DCM cohort, the clinical endpoint committee could classify an event as a VT based on available information from hospital records or other sources.

DANISH used an endpoint classification committee which used prespecified criteria to adjudicate all prespecified clinical outcomes (more details in their Supplemental Appendix). The national social security death index database was queried for vital status if appropriate. DCM-VT received a preprogrammed database from each contributing center. In this database, if a patient died, the center had to enter a cause of death. The options were prespecified. Free text entry was also possible. The data sets were merged by the primary investigating centre (LUMC). Follow-up was capped at 5 years for all analyses.

### Predefined subgroups

#### Propensity-matched cohorts

Patients from VT-DCM and P-DCM cohorts were matched based on age, sex, LVEF, and estimated glomerular filtration rate (eGFR) and heart-failure medication (angiotensin-converting enzyme [ACE] inhibitor/angiotensin II receptor blockers, betablockers, and mineralocorticoid receptor antagonists [MRAs]). These variables were selected based on their well-established association with mortality in heart failure.^8^, ^9^, ^10^ As the P-DCM cohort only included patients with LVEF ≤35%, matching with patients from the VT-DCM cohort was restricted to those with an LVEF ≤35%.

#### VT-DCM patients with VT ablation within 1 year after first sustained VT

This subgroup consists of patients from the VT-DCM cohort in whom data on the first sustained VT were available and who were scheduled for early VT ablation, defined as ablation within less than 1 year after the first presentation with any sustained VT. This subgroup was predefined because there may be a selection bias due to a higher expected benefit from early ablation in young patients with less comorbidity.

#### P-DCM patients with sustained VT during follow-up

To evaluate outcomes after the first sustained VT episode, a separate analysis was performed with follow-up starting immediately after this VT episode. This allows comparison of P-DCM patients with VT to VT-DCM patients with VT, where we hypothesized that there would be no significant difference in outcomes.

### Statistics

The baseline characteristics between the 2 cohorts were compared using chi-square or Wilcoxon tests, as appropriate. A Cox proportional hazards model was created to detect significant predictors of the primary and secondary outcomes and reported as unadjusted and adjusted HRs (aHRs) with 95% CI. Multiple chained imputation was used to estimate missing variables (4% missing, number of imputations = 4). Propensity score matching using nearest neighbor was performed between the P-DCM and VT-DCM study cohorts, aiming to achieve a standardized mean difference of <0.2 for the matched variables. Kaplan-Meier plots were constructed for the composite of total mortality or heart transplantation. Two-sided *P* values of 0.05 or less were considered statistically significant. The following variables which are known to be associated with mortality and which were prospectively collected in both cohorts were selected for univariable analysis: age, sex, comorbidities (chronic obstructive pulmonary disease [COPD], diabetes mellitus [DM], hypertension [HT], stroke, and atrial fibrillation [AF]), CRT, kidney function (eGFR), LVEF, NYHA functional class, QRS width and medication use (ACE inhibitors/angiotensin receptor blockers [ARBs], amiodarone, beta-blocker, MRAs, and statins). Variables found to be significant on univariable analysis at *P* < 0.10 were subsequently included in the multivariable analysis. To estimate the impact of the occurrence/recurrence of VT on the risk of NSCVD (secondary outcome), we performed a landmark analysis (Supplemental Table 4). Competing risks were assessed using a Fine-Gray analysis (Supplemental Figure 5). Event rates were expressed as events per 100 patient-years. To assess whether differences in event rates were statistically significant, the incidence rate ratios were calculated followed by a z-test. Analyses were performed using R software (version 4.0.4, R Project for Statistical Computing).

## Results

Table 1 shows the characteristics of the merged group (by cohort) and of the matched subgroups. A total of 828 patients (median age 64 years, 23.2% females) were included. The median LVEF at inclusion was 26% (IQR: 20%-33%). The majority of the patients had a NYHA class of I or II (59.9%), and almost all patients had an ICD at baseline (98.3%). Of these, 55.1% had a CRT device.

**Table 1:** Patient Characteristics and Outcomes

	Overall (N = 828)	All Patients by Cohort			After Matching		
		P-DCM (n = 556)	VT-DCM (n = 272)	*P* Value	P-DCM (n = 142)	VT-DCM (n = 142)	*P* Value
Age, y	64 [55-71]	64 [56-72]	62 [51-71]	0.024	64 [54-71]	64 [53-71]	0.988
Female	192 (23.2)	151 (27.2)	41 (15.1)	<0.001	19 (13.4)	19 (13.4)	1.000
COPD	75 (9.1)	55 (10.0)	20 (7.6)	0.333	12 (8.5)	11 (7.7)	0.700
DM	150 (18.1)	99 (17.8)	51 (19.5)	0.634	33 (23.2)	38 (26.8)	0.584
HTN	302 (36.5)	181 (32.6)	121 (46.4)	<0.001	49 (34.5)	71 (50.0)	0.001
Stroke	59 (7.3)	52 (9.4)	7 (2.7)	0.001	16 (11.3)	4 (2.8)	0.002
AF	337 (41.1)	237 (42.6)	100 (38.0)	0.240	71 (50.0)	70 (49.3)	1.000
eGFR <50 mL/min/1.73 m^2^	177 (21.4)	94 (16.9)	83 (31.3)	<0.001	62 (43.7)	59 (41.5)	0.810
LVEF (%)	26 [20-33]	25 [20-30]	35 [27-43]	<0.001	28 [24-32]	29 [23-33]	0.644
NYHA functional class				<0.001			0.054
I/II	297 (53.4)	297 (53.4)	199 (73.2)		75 (52.8)	92 (64.8)	
III/IV	259 (46.6)	259 (46.6)	61 (22.4)		67 (47.2)	50 (35.2)	
QRS width (ms)	140 [108-164]	146 [114-166]	114 [100-144]	<0.001	144 [114-166]	130 [105-151]	0.048
ACEI/ARB	745 (90.0)	533 (95.9)	212 (78.5)	<0.001	122 (85.9)	117 (82.4)	0.516
Amiodarone	160 (19.3)	34 (6.1)	126 (47.5)	<0.001	8 (5.6)	78 (54.9)	<0.001
BB	738 (89.1)	509 (91.5)	229 (84.5)	0.003	127 (89.4)	127 (89.4)	1.000
MRA	432 (52.2)	326 (58.6)	106 (39.0)	<0.001	79 (55.6)	77 (54.2)	0.905
Statin	330 (39.9)	242 (43.5)	88 (32.5)	0.003	75 (52.8)	49 (34.5)	0.006
CRT	440 (55.1)	323 (59.2)	117 (46.4)	0.001	76 (53.5)	73 (51.4)	0.496
ICD	814 (98.3)	556 (100)	258 (94.9)	-	142 (100)	139 (97.9)	-
Outcomes							
Death/HTx	148 (17.9)	3.96/100py	11.10/100py	<0.01	3.63/100py	18.37/100py	<0.001
NSCVD	78 (9.4)	1.87/100py	6.82/100py	<0.01	1.36/100py	12.68/100py	<0.001
Non-CV death	40	1.39/100py	1.56/100py	-	-	-	-

Values are n (%) or median [IQR]. Outcomes are per 100 person-years (py).

ACEI = angiotensin-converting enzyme inhibitor; AF = atrial fibrillation; ARB = angiotensin receptor blocker; BB = beta blocker; COPD = chronic obstructive pulmonary disease; CRT = cardiac resynchronization therapy; CV = cardiovascular; DM = diabetes mellitus; eGFR = estimated glomerular filtration rate; HTN = hypertension; HTx = heart transplantation; ICD = implanted defibrillators; LVEF = left-ventricular ejection fraction; MRA = mineralocorticoid receptor antagonist; NSCVD = nonsudden cardiovascular death.

**Figure 1:**
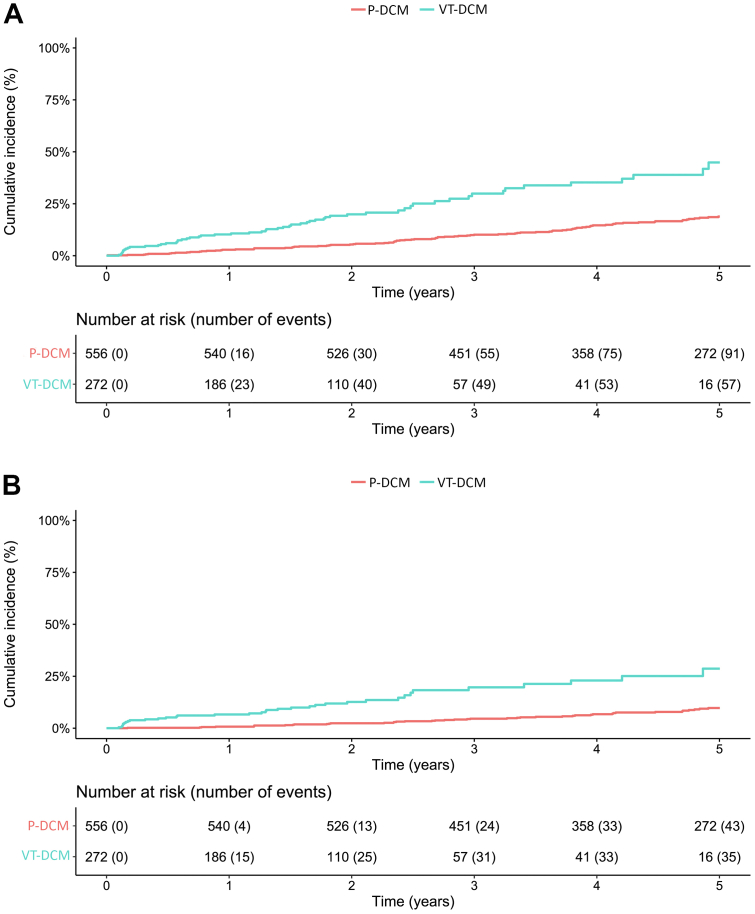
Primary and Secondary Outcomes (A) Primary endpoint of the composite of mortality or heart transplantation. Univariate Cox regression: HR: 3.11 (95% CI: 2.21-4.39); *P* < 0.001. (B) Secondary endpoint NSCVD. Univariate Cox regression: HR: 4.10 (95% CI: 2.58-6.52); *P* < 0.001. DCM = dilated cardiomyopathy; NSCVD = nonsudden cardiovascular death; P-DCM = primary prevention DCM cohort; VT = ventricular tachycardia; VT-DCM = DCM cohort which experienced VT and subsequent ablation.

During a median follow-up of 3.9 years (IQR 2.1-5.8), the composite endpoint of all-cause mortality and heart transplantation was reached in 148/828 patients (17.9%). In the P-DCM cohort, 556 patients were followed for a median of 4.9 years (IQR: 3.4-6.6). Within the first 5 years of follow-up, 91 patients (16.4%) died. No patient underwent heart transplantation. The incidence of all-cause mortality was 3.96 (95% CI: 3.19-3.86) per 100 person-years. Noncardiovascular death occurred in 32 patients (35.2% of all deaths). In the VT-DCM cohort, 272 patients were followed for a median of 1.8 years (IQR: 0.6-2.5). Of these, 57 patients (21%) experienced the composite primary outcome of mortality and heart transplantation within 5 years (Figure 1A). Nine patients underwent heart transplantation. The incidence of mortality and heart transplantation was 11.10 (95% CI: 8.41-14.39) per 100 person-years. Noncardiovascular death occurred in only 8 patients (14.0% of all deaths). Early occurrence/recurrence of VT (within 31 days) appears to raise the risk of the primary endpoint further (unadjusted HR: 6.34 [95% CI: 3.63-11.06]).

In univariable analysis, the composite of mortality or heart transplantation was associated with history of VT at baseline, age (per year increase), sex, COPD, history of DM, history of AF, eGFR <50 mL/min/1.73 m^2^, NYHA functional class III/IV, and amiodarone use. The association of LVEF (per % increase) did not reach statistical significance.

**Figure 2:**
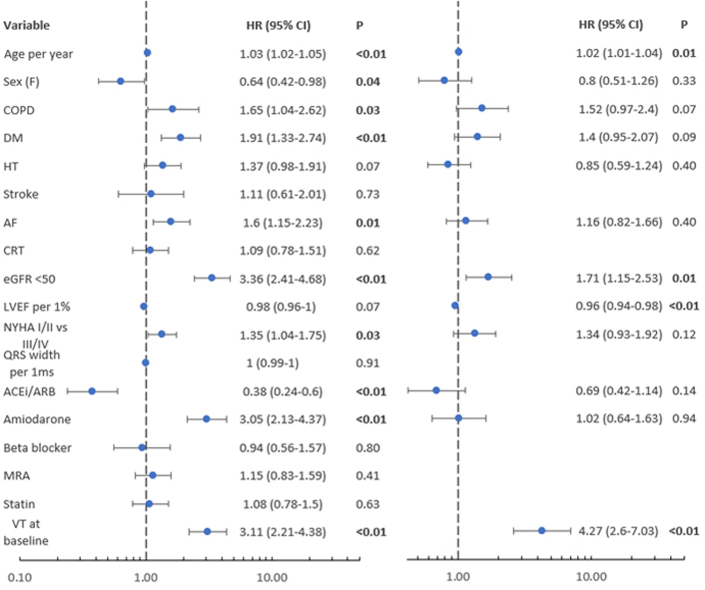
Univariable and Multivariable Analyses for the Primary Endpoint Multivariable analysis performed with univariable predictors with *P* value < 0.10. Significant variables are bold *P* values. eGFR unit mL/min/1.73 m^2^. ACEi = angiotensin-conversion enzyme inhibitors; AF = atrial fibrillation; ARB = angiotensin receptor blocker; COPD = chronic obstructive pulmonary disorder; CRT = cardiac resynchronization therapy; DM = diabetes mellitus; eGFR = estimated glomerular filtration rate; HT = hypertension; LVEF = left-ventricular ejection fraction; MRA = mineralocorticoid receptor antagonist; other abbreviations as in Figure 1.

In multivariable analysis the composite of mortality or heart transplantation was associated with history of VT at inclusion (aHR: 4.27 [95% CI: 2.60-7.03]; *P* < 0.01), age (per year increase, aHR: 1.02 [95% CI: 1.01-1.04]; *P* = 0.01), eGFR <50 mL/min/1.73 m^2^ (aHR: 1.71 [95% CI: 1.15-2.53]; *P* = 0.01), and LVEF (per % increase, aHR: 0.96 [95% CI: 0.94-0.98]; *P* < 0.01) (Figure 2).

### Nonsudden cardiovascular death

NSCVD occurred in 43 (7.7%) patients in the P-DCM cohort and in 35 (12.9%) patients in the VT-DCM cohort (Figure 1B). NSCVD was due to progression to end stage heart failure in the majority of VT-DCM patients (63% of NSCVD). In the P-DCM cohort, the proportion of NSCVD due to progressive heart failure was not available. Early occurrence/recurrence of VT (within 31 days) appears to also raise the risk of the secondary endpoint further (unadjusted HR: 10.89 [95% CI: 5.82-20.39]).

In univariable analysis NSCVD was associated with VT at inclusion, age (per year increase), history of DM, history of HT, AF, eGFR <50 mL/min/1.73 m^2^, LVEF (per % increase), NYHA functional class III/IV, ACE inhibitor/ARB use, and amiodarone use (Supplemental Figure 1).

In multivariable analysis, NSCVD was associated with VT at inclusion (aHR: 7.28; 95% CI: 3.80-13.97; *P* < 0.01), eGFR <50 mL/min/1.73 m^2^ (aHR: 2.10; 95% CI: 1.22-3.62; *P* = 0.01), LVEF (per % increase. aHR: 0.94; 95% CI: 0.92-0.97; *P* < 0.01), and NYHA functional class III/IV (aHR: 1.82; 95% CI: 1.09-3.02; *P* = 0.02) (Supplemental Figure 1).

### Propensity-matched cohorts

Before matching, the VT-DCM cohort was younger, had on average a higher ejection fraction, a lower NYHA class, and less heart-failure medications (Table 1). The proportion of patients with reduced GFR at admission for CA was higher in the VT-DCM cohort. VT-DCM patients had a significantly narrower QRS complex and a lower percentage of patients were discharged with CRT. After matching, a standardized mean difference of <0.2 was achieved for all covariates matched (Supplemental Figure 2). CRT was no longer significantly different between the groups. Amiodarone use remained higher in the VT-DCM group.

### Primary and secondary outcomes after propensity matching

**Figure 3:**
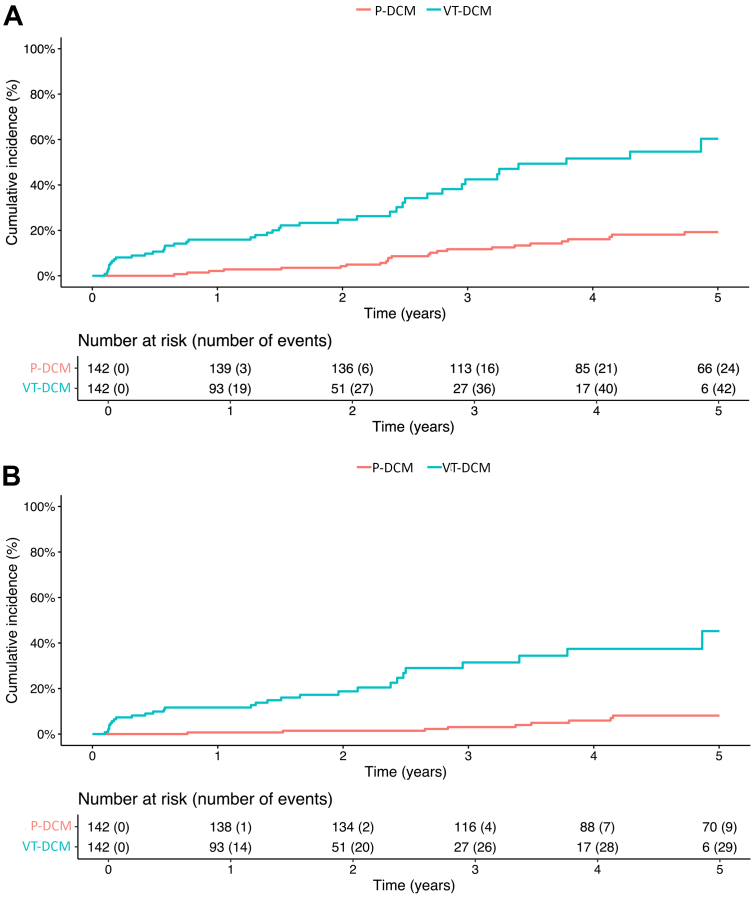
Primary and Secondary Outcomes in Matched Cohorts (A) Primary outcome for the matched cohorts. Univariate Cox regression: HR: 4.43 (95% CI: 2.64-7.43); *P* < 0.01. (B) Secondary outcome of NSCVD for the matched cohorts. Univariate Cox regression: HR: 7.10 (95% CI: 4.34-11.61); *P* < 0.01. Abbreviations as in Figure 1.

For the matched sample the outcome of 142 patients of the VT-DCM cohort were compared with 142 patients of the P-DCM cohort. The matched cohort was followed up for a median of 2.8 years [IQR: 1.4-4.8]. In the matched P-DCM cohort, 24 patients (16.9%) experienced the primary outcome giving an incidence rate of the primary outcome of 3.63 per 100 person-years. In the matched VT-DCM cohort, 42 patients (29%) experienced the primary outcome with an incidence rate of 18.37 per 100 person-years. This gives an incidence rate ratio of 5.06 (95% CI: 3.06-8.36; *P* < 0.001). In the matched cohorts, history of VT at inclusion was associated with an increased risk of the composite of mortality or heart transplantation (HR: 4.43 [95% CI: 2.64-7.43]) (Figure 3A).

Within the matched P-DCM cohort, 9 patients (6.3%) experienced the secondary outcome of NSCVD. Within the matched VT-DCM cohort, 29 patients (20.4%) experienced NSCVD.

In the matched cohorts, history of VT at inclusion was associated with an increased risk of NSCVD (HR: 7.10 [95% CI: 4.34-11.61]) (Figure 3B).

### VT-DCM patients with VT ablation within 1 year after first sustained VT

**Figure 4:**
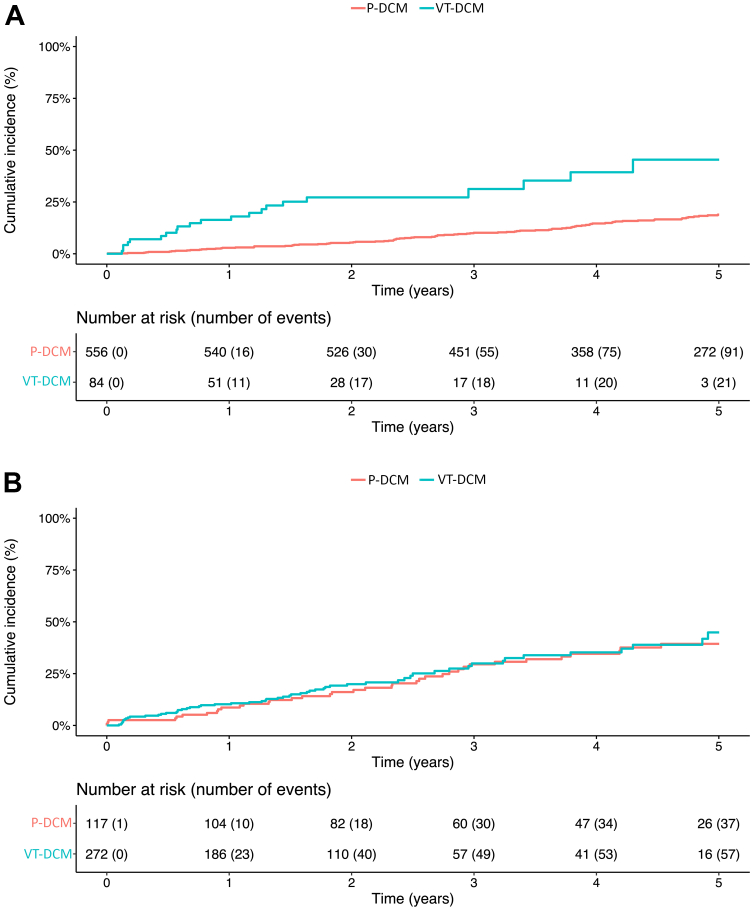
Outcomes in Subgroups (A) Primary outcome in VT-DCM patients with early ablation vs P-DCM. Early ablation refers to within 1 year. Univariate Cox regression: HR: 4.07 (95% CI: 2.50-6.61); *P* < 0.01. (B) Primary outcome in P-DCM patients *after* first VT vs VT-DCM. P-DCM patients who had sustained VT during follow-up were included from the moment of their sustained VT episode. Univariate Cox regression: HR: 1.12 (95% CI: 0.73-1.70); *P* = 0.61. Abbreviations as in Figure 1.

Of the VT-DCM cohort, 84 patients (31%) underwent ablation within 1 year of their first presentation with VT. Baseline characteristics are displayed in Supplemental Table 2. There were no significant differences between patients who underwent VT ablation within 1 year and after 1 year after the first event, apart from incidence of HT. This VT-DCM subgroup was followed for a median of 1.4 years [IQR: 0.3-2.2] and 21 of them (25%) experienced the primary outcome. Compared to the P-DCM cohort (Figure 4A) patients with VT had greater cumulative mortality or heart transplantation (HR: 3.57 [95% CI: 1.98-6.46, *P* < 0.01]) (adjusted for age, sex, eGFR, history of diabetes and AF, NYHA functional class, and use of amiodarone and ACE/ARB) and NSCVD (HR: 14.80 [95% CI: 6.51-33.64; *P* < 0.01]) (after adjusting for age, LVEF, eGFR,history of diabetes and AF, NYHA functional class, and use of amiodarone, beta-blocker, and ACE/ARB) (Supplemental Figure 3).

### VT after a primary prevention ICD

Of the P-DCM cohort, 117 patients (84% male) experienced an episode of sustained VT during follow-up at a median age of 62 years. Baseline characteristics of these patients are provided in Supplemental Table 3. The median time to sustained VT was 4.4 years (IQR: 2.8-6.0) after randomization. These patients were then followed up to 5 years after their sustained VT.

Patients with VT after primary prevention ICD had similar outcomes to the patients with VT at inclusion from the VT-DCM cohort with regards to all-cause mortality (VT-DCM HR: 1.33 [95% CI: 0.69-2.55]; *P* = 0.39) (adjusted for age, LVEF, eGFR, history of COPD, diabetes and AF, NYHA functional class, and use of amiodarone and MRA) (Figure 4B) and NSCVD (VT-DCM aHR: 1.46 [95% CI: 0.64-3.32]; *P* = 0.37) (adjusted for LVEF, eGFR, history of AF, NYHA functional class, and use of amiodarone and MRA) (Supplemental Figure 4).

## Discussion

We found that in patients with NICM, the occurrence of spontaneous monomorphic sustained VT is a strong predictor of all-cause mortality and NSCVD, independent of other clinical predictors of mortality commonly associated with the severity of heart failure (Central Illustration).

**Central Illustration:**
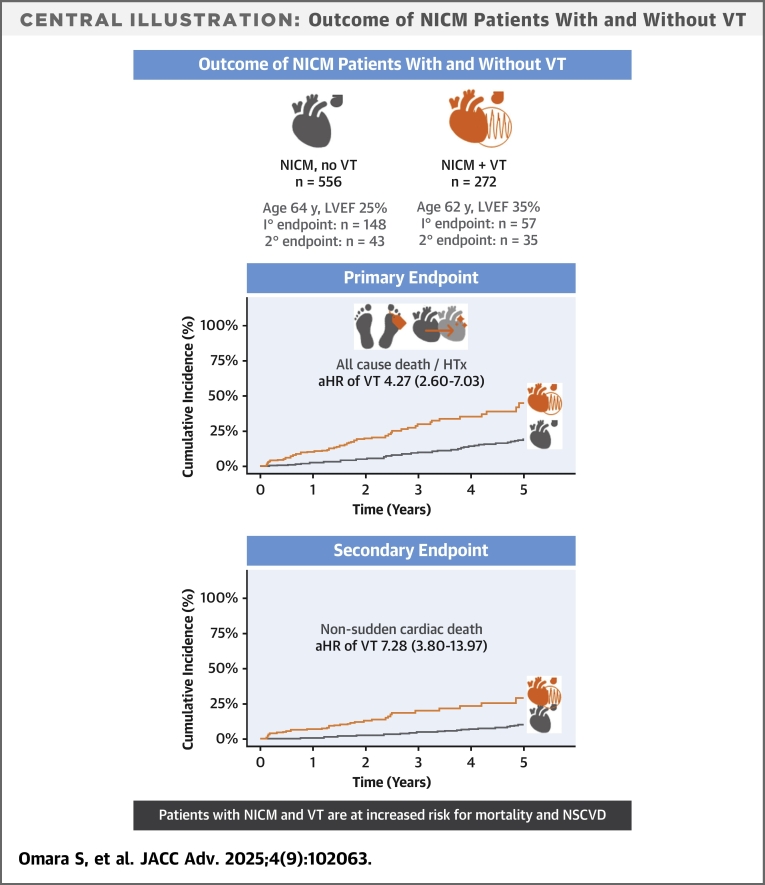
Outcome of NICM Patients With and Without VT Outcomes for the primary prevention cohort (P-DCM) and the VT cohort (VT-DCM). Incidence of both the primary and secondary endpoints was significantly higher in the VT-DCM cohort, which was confirmed in the mentioned statistical methods. This implies that patients with nonischemic cardiomyopathies are at increased risk for death from rapidly progressive heart failure. DCM = dilated cardiomyopathy; HTx = heart transplantation; LVEF = left-ventricular ejection fraction; NICM = nonischemic cardiomyopathy; NSCVD = nonsudden cardiovascular death; VT = ventricular tachycardia.

All-cause and nonsudden cardiovascular mortality were greater among NICM patients referred for VT ablation, who were discharged for routine follow-up in the outpatient setting, than among stable NICM patients randomized to ICD implantation, despite a lower LVEF and older age in the latter group. This finding was consistent for VT-DCM patients who were referred for ablation soon after their first sustained VT and even more pronounced in propensity-matched cohorts. Importantly, a similar worse prognosis was also observed in the primary prevention ICD group after their initial episode of VT, further consolidating the evidence. Cardiovascular mortality was mainly due to progressive heart failure in VT-DCM patients and not due to VT. These data suggest that the occurrence of VT in NICM may indicate a myocardial disease substrate that is associated with a worse prognosis and accelerated disease progression.

### Prediction of all-cause mortality and nonsudden cardiac mortality

Prognostication in NICM patients is important for counseling, treatment planning, and to refer patients to centers capable of providing advanced heart failure treatment. Several risk markers and scores have been developed to predict all-cause mortality, cardiovascular mortality, or hospitalization. Suggested candidate predictors from large trials derived from mixed cohorts of patients with heart failure with reduced ejection fraction include age, sex, prolonged QRS duration, AF, NYHA class, LVEF, extracardiac vascular disease, and noncardiovascular comorbidity (DM, CKD, and COPD).^11^^,^^12^ To our knowledge, there are no prediction models specifically for NICM patients. In our merged NICM cohort, variables associated with mortality in univariable analysis were age, sex, NYHA functional class, LVEF, AF, eGFR <50 (mL/min/1.73 m^2^), comorbidities (ie, DM and COPD), and a prior episode of sustained VT. Age, LVEF, eGFR <50, and prior sustained VT remained independently associated with all-cause mortality and NSCVD. At inclusion, patients in the P-DCM cohort were older, had a lower LVEF, higher NYHA functional class and wider QRS complexes, risk factors that would suggest a higher total mortality and NSCVD compared to the VT-DCM population. However, in all of our analyses, all-cause and cardiovascular mortality were significantly higher in patients with VT. Notably, after propensity matching, the HR of prior sustained VT increased for both all-cause mortality and NSCVD. This implies that the higher LVEF and lower NYHA functional class of the VT-DCM cohort, among other factors, may mask an even larger effect size of prior sustained VT. In this context, it is also worth noting the difference in noncardiovascular mortality, which was much higher in the primary prevention cohort (35%) vs the VT cohort (14%). In addition, the aHRs for the secondary outcome of NSCVD was much greater than it was for all-cause death (namely 7.28 vs 4.27). This finding suggests that heart failure death is a major contributor to the increased mortality in patients with VT.

### Benefit of primary prevention ICDS in NICM—a temporary gain?

In the DANISH trial, the risk of SCD was halved with an ICD, yet there was no overall long-term survival benefit. All time-to-event curves appear to diverge during the initial 5 years of the trial only to subsequently converge again, as can also be gleaned from their post hoc analysis of annual event rate ratios. These findings are consistent with observations made during the long-term follow-up of NICM patients in SCD-HeFt (Sudden Cardiac Death in Heart Failure Trial).^13^ Time-variable analysis showed that the early phase was associated with an incremental benefit for ICD therapy in NICM. However, after 6 years, mortality rates (ICD vs placebo) converged with a cumulative event rate of 45.1% vs 44.0% after 10 years.^14^

Patients who experience a first episode of sustained VT benefit from the ICD during this event. However, if patients with VT have more rapidly progressive heart failure after the first event, the initial gain is lost, which may explain the converging curves. This assumption is further supported by our observation that the long-term estimates of mortality were similar following a first VT episode for primary prevention patients compared with patients with a history of VT.

### ICD therapy or VA as determinant of mortality

ICD shocks have been associated with mortality in the SCD-Heft trial and it has been suggested that ICD shocks directly contribute to mortality. However, neither defibrillation testing^15^ nor ICD shocks for supra-VT or oversensing in the absence of spontaneous VA^16^ have been associated with mortality. These data suggest that the increased risk is predominantly related to the VA and related substrate. Data regarding the association between anti-tachycardia pacing (ATP) and mortality are conflicting. A meta-analysis of 4 studies, including predominantly (87%) patients with ICM reported no association between ATP and mortality.^17^ However, in 2 more recent studies, including a larger proportion of patients with NICM, ATP was independently associated with mortality.^18^^,^^19^

### Nonischemic fibrosis: substrate of VT

Monomorphic sustained VT in NICM is most often due to scar-related reentry with myocardial fibrosis creating the arrhythmia substrate.^20^ Different types of nonischemic fibrosis have been described, including replacement fibrosis, interstitial fibrosis, and perivascular fibrosis and the extent of these can vary. Late gadolinium enhancement (LGE) on cardiac magnetic resonance imaging likely indicates replacement fibrosis, which can be a progressive pathological process. Both presence and progression of LGE were independent predictors of mortality during a median follow-up of 4.8 years in a recent cohort of DCM patients who underwent serial cardiac magnetic resonance imaging.^21^ LGE-cardiac magnetic resonance imaging does not provide a complete picture. Less confluent and less dense fibrosis does not reliably manifest as LGE.^22^ In genetic NICM due to nondesmosomal mutations, the presence and extent of LGE were not related to regional or global left ventricular function.^23^ The occurrence of VT may reflect areas with larger amounts of confluent diffuse fibrosis, essentially the “tip of the iceberg” in a ventricle that is already more globally diseased, than is suggested by the degree of left ventricular dysfunction. Even a relatively modest further increase in fibrosis may constitute a threshold event, beyond which a more rapid decline in systolic function ensues.

### Study Limitations

Despite prospective data collection, the analyses reported in this study remain observational and causality cannot be completely inferred. Despite adjustment for comorbidities and differences in baseline characteristics, residual confounding cannot be excluded. The degree of heterogeneity of etiologies of nonischemic cardiomyopathies between the 2 cohorts is unclear, although there are ameliorative factors to consider, as discussed earlier. Although the current data support VT as a strong marker for the occurrence of nonsudden death, the reasons for this association may be complex and could include the contribution of therapies used to treat VT in the hope of preventing recurrences, including antiarrhythmic drug therapy, alterations in conduction induced by therapy, and adverse effects of CA. Even if adverse effects of therapy for arrhythmia contribute to poor outcomes in some patients, they are not completely avoidable because treatment to attempt to prevent VT is needed. Despite the differences in study design, the availability of 2 large contemporary cohorts of patients with NICM and either a documented sustained VT or the absence of any prior sustained VT at the time of inclusion and the availability of patient-level data may justify the analysis.

## Conclusions

Patients with NICM experiencing sustained VT are at an increased risk for mortality and NSCVD, independent of other predictors such as the LVEF. This suggests that a sustained VT could be a marker for a more severe, rapidly progressive, cardiomyopathy. This may have important clinical implications, such as risk stratification and potentially escalation of heart failure therapies.

Perspectives **COMPETENCY IN MEDICAL KNOWLEDGE:** In NICM, sustained VT signals advanced disease and elevated risk of both arrhythmic and nonarrhythmic death. Patients referred for early VT ablation had a substantially greater risk of NSCVD, which is likely related to progressive heart failure. This recognition can aid in risk stratification and consideration for escalation of heart failure therapies (e.g., intensive monitoring, left-ventricular assist device, and heart transplantation). This could maintain the survival benefit of an ICD.

**TRANSLATIONAL OUTLOOK:** Our findings may contribute to the ongoing discussion on the survival benefit from ICDs for primary prevention of SCD in patients with NICM. Linking arrhythmic events to heart failure management pathways may improve outcomes in NICM. Randomized trials are needed to determine if this strategy translates into improved long-term survival in this high-risk population.

## Funding support and author disclosures

Supported by unrestricted grants from 10.13039/100004374Medtronic, 10.13039/100006279St. Jude Medical, 10.13039/501100007437TrygFonden, and the 10.13039/100007405Danish Heart Foundation. This study was partially supported by an investigator-initiated grant (IIS-310) from 10.13039/100007497Biosense Webster (a Johnson & Johnson company). Dr Koeber reports receiving lecture fees from Sanofi and Novartis. Dr Thune, lecture fees from Bristol-Myers Squibb and personal fees and travel support from Novartis. Dr Pehrson, lecture fees from Bayer, Boehringer Ingelheim, Bristol-Myers Squibb, and Servier. Dr Berruezo is a stockholder in ADAS3D; and has received research grants from 10.13039/100007497Biosense Webster and 10.13039/501100005035Biotronik. Dr Vaseghi is an educational consultant for Biosense Webster; and holds founder shares in NeuCures. Dr Wijnmaalen has received research grants from 10.13039/100007497Biosense Webster to the Department of Cardiology at Leiden University Medical Center. Dr Stevenson has received speaker honoraria from Abbott, Boston Scientific, Biotronik, Johnson & Johnson, and Medtronic; and has received a research grant from Thermedical. Dr Nielsen has received consulting fees from Boston Scientific and lecture fees from Biosense Webster and Biotronik. All other authors have reported that they have no relationships relevant to the contents of this paper to disclose.
