# Supplementary material for: Ventricular Tachycardia Predicts All-Cause Mortality and Nonsudden Cardiac Death in Nonischemic Cardiomyopathy
Source: JACC Adv. 2025 Aug 13;4(9):102063. doi: 10.1016/j.jacadv.2025.102063 (PMC12362670; doi:10.1016/j.jacadv.2025.102063)
Supplement: Supplementary data [file mmc1.docx]

**SUPPLEMENTAL APPENDIX**

Supplemental Figure 1. Univariable and multivariable analyses for the secondary endpoint of NSCVD (all patients).

*
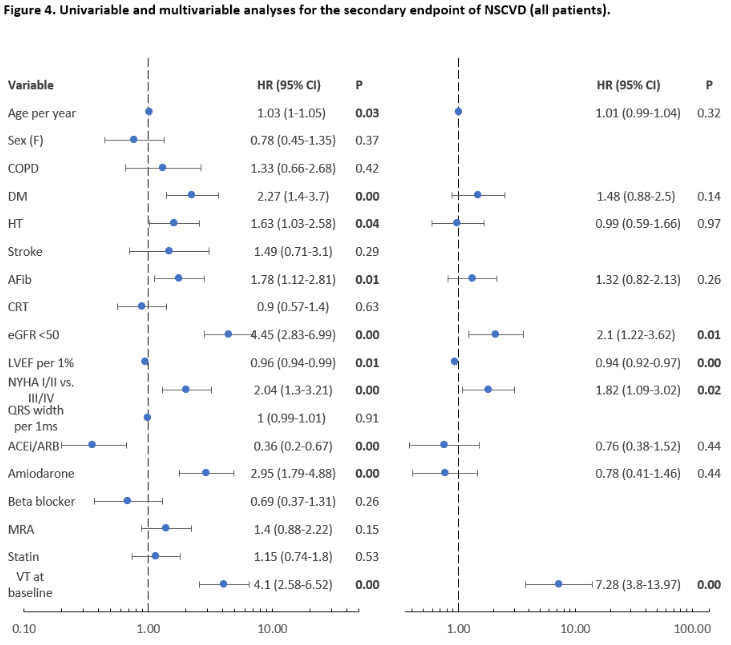
*

*Multivariable analysis performed with univariable predictors with P value <0.10. Significant variables are bold p-values. eGFR unit ml/min/1.73m2.*

Supplemental Figure 2. Standardised mean difference matching. P-DCM in red, VT-DCM in cyan.


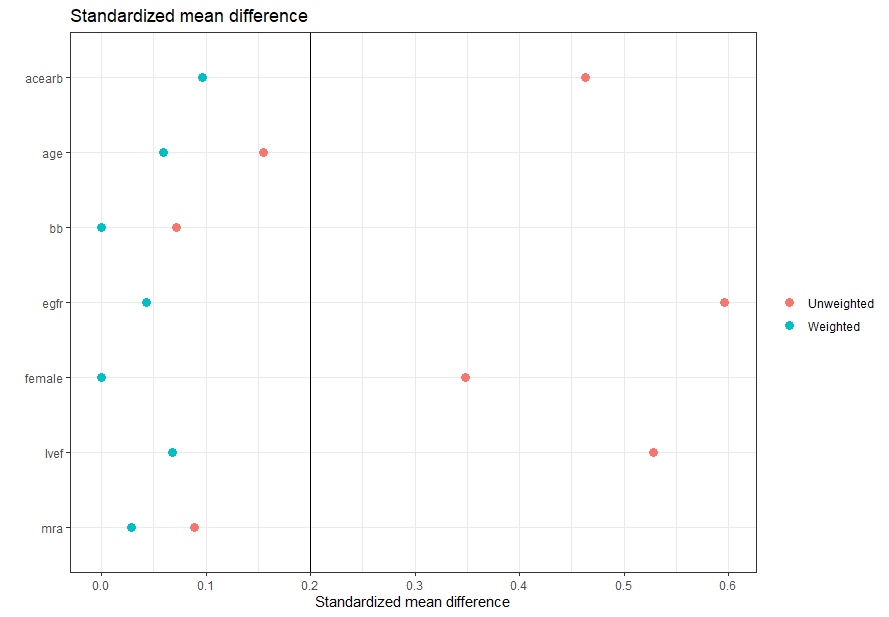


Supplemental Figure 3. Cumulative incidence of the secondary outcome of NSCVD in VT-DCM patients with early ablation for a sustained VT (within one year after first VT) vs. P-DCM cohort.

*
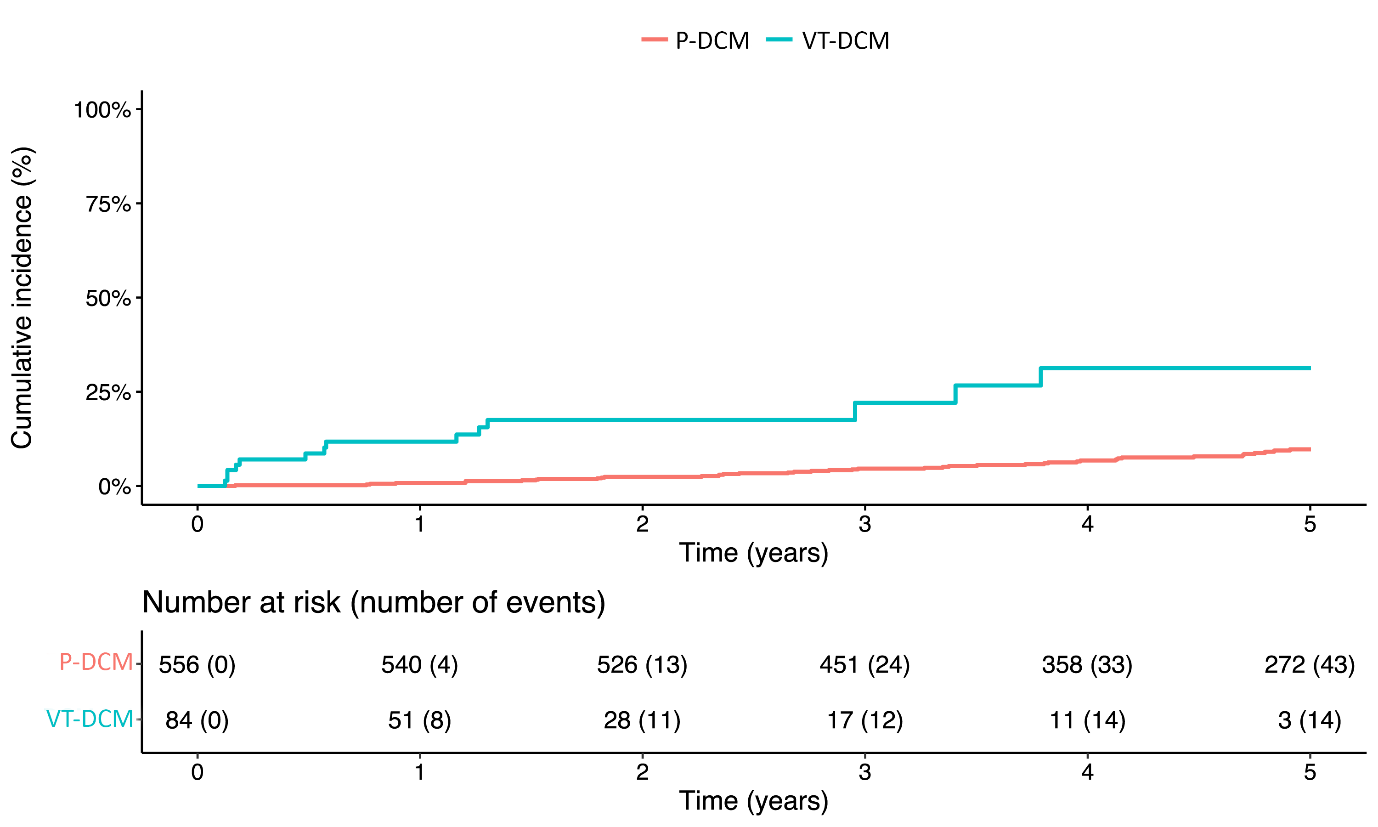
*

*Univariate Cox regression: HR 5.96 [95%CI: 3.20-11.10]; P < 0.01.*

Supplemental Figure 4. Cumulative incidence of NSCVD in the P-DCM cohort after experiencing their first VA vs. in the VT-DCM cohort.


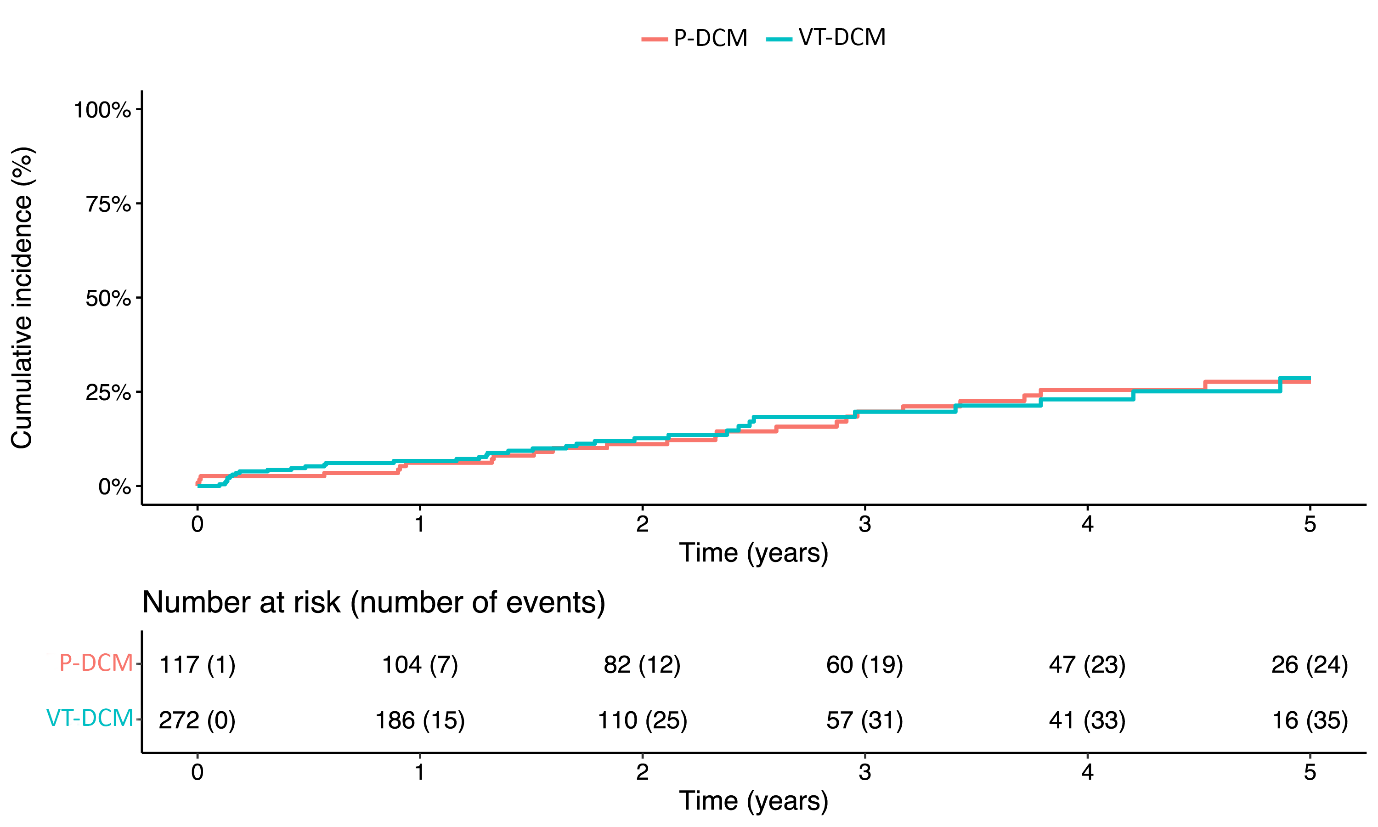


*Univariate Cox regression: HR 1.03 [95%CI: 0.61-1.75]; P = 0.90.*

### Competing risk analysis:

Patients in our study population are at risk for multiple types of death simultaneously, which necessitates appropriate statistical methods.

In our analysis, we estimated the risk of non-sudden cardiovascular death adjusted for the competing risk of other types of death using the Fine-Gray method. Our results show that the VT-DCM group had a significantly higher subdistribution hazard ratio of 3.15 (95% CI: 2.10-4.73) compared to the P-DCM cohort, when accounting for the competing risk of death due to other causes.

| Group | Patients (n) | Events | Unadjusted |
| --- | --- | --- | --- |
| P-DCM | 556 | 185 | Reference |
| VT-DCM | 272 | 81 | 3.15 (2.10 - 4.73) |

Supplemental figure 5. Competing risk analysis: non-sudden CV death and other types of death.

*
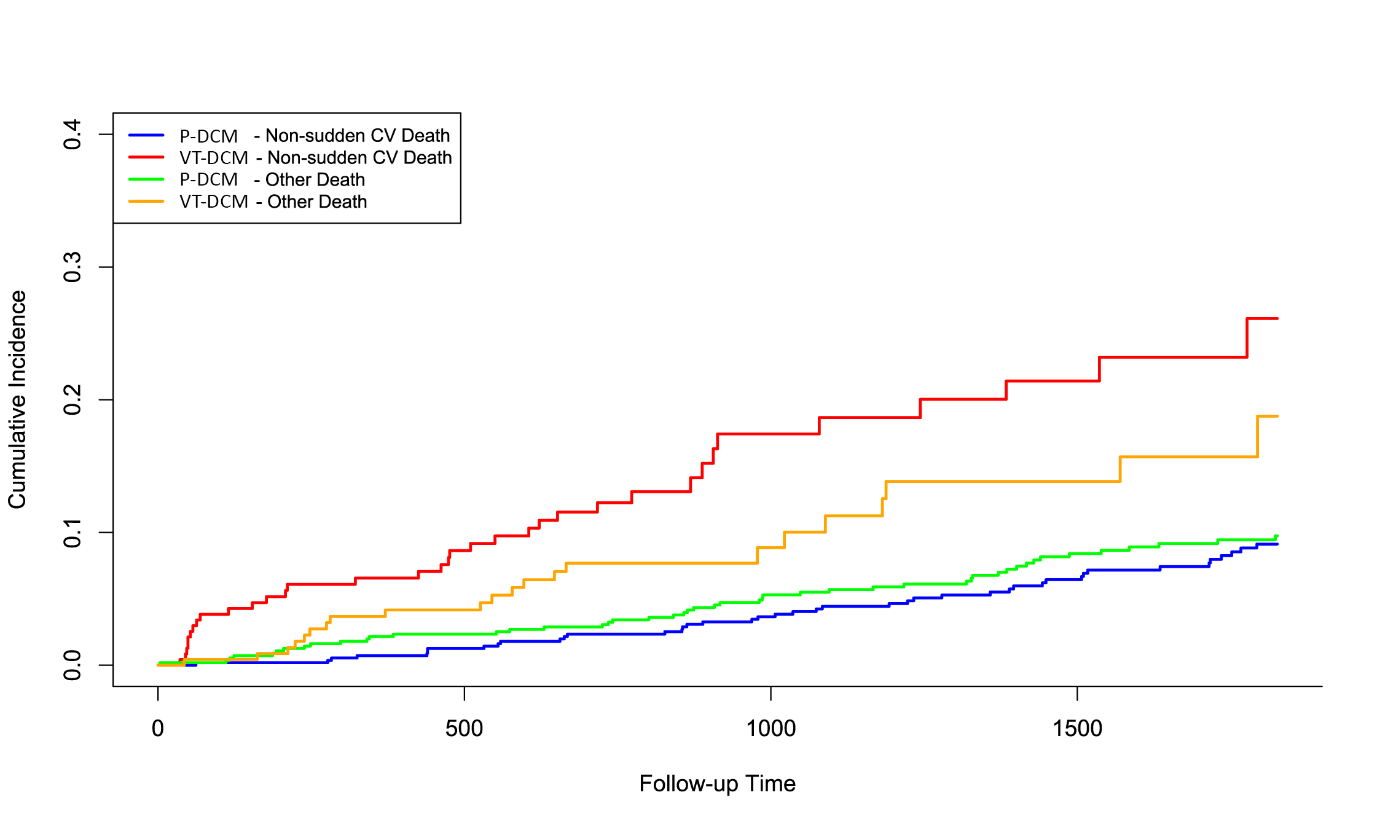
*

### Differences between cohorts:

Supplemental Table 1 shows similarities and differences between the studies with regards to in- and exclusion criteria.

Supplemental Table 1. In- and exclusion criteria for DANISH and DCM-VT.

|  | Shared | DANISH only | DCM-VT only |
| --- | --- | --- | --- |
| Inclusion | - Non-ischaemic aetiology - Informed consent | - Optimal medical treatment - Clinical heart failure - NYHA II or III, or IV if CRT was planned - NT-pro BNP >200pg/ml | - **Ventricular tachycardia (accepted for ablation)** |
| Exclusion | - Uncorrected congenital heart disease - Uncorrected valve obstruction - Dysregulated permanent atrial fibrillation (AF) or paroxysmal AF without recent rapid tachycardia (DANISH)/tachycardia-induced cardiomyopathy (DCM-VT) - Active myocarditis - Restrictive cardiomyopathy - Hypertrophic/obstructive cardiomyopathy - Renal failure treated with dialysis - Current malignancy - Adrenal insufficiency - Recipient of any major organ transplant - Active vasculitis | - Untreated thyroid disease - Recent chemo- or radiotherapy for malignancy - Recent history of alcohol/illicit drug abuse disorder - Any condition (e.g. psychiatric illness) that could put subject at significant risk, confound the study results or interfere significantly with subject’s participation in the study | - Corrected congenital heart disease - Prior valve replacement - Past myocarditis - Right-dominant cardiomyopathy - LV noncompaction - Chagas disease - Cardiac sarcoidosis - Urgent waiting list for heart transplant |

The shared criteria have a green font, the differences are in red [except for the independent variable (VT)].

### Baseline characteristics based on ablation time in VT-DCM:

Supplemental Table 2. Baseline characteristics of patients that underwent a VT ablation <1 year or >=1 year after the first VT occurrence.

|  | | **Overall*** | **<one year ablation** | **≥one year ablation** | **P value** |
| --- | --- | --- | --- | --- | --- |
| **N** | | 272 | 84 | 173 |  |
| **Age** | | 62 [51, 71] | 64 [56, 71] | 62 [49, 70] | 0.404 |
| **Female** | | 41 (15.1) | 15 (17.9) | 23 (13.3) | 0.436 |
| **COPD** | | 20 (7.4) | 7 (8.3) | 13 (7.5) | 0.547 |
| **DM** | | 51 (18.8) | 18 (21.4) | 31 (17.9) | 0.237 |
| **HT** | | 121 (44.5) | 51 (60.7) | 64 (37.0) | <0.001 |
| **Stroke** | | 7 ( 2.6) | 2 (2.4) | 4 (2.3) | 0.900 |
| **AFib** | | 100 (36.8) | 30 (35.7) | 65 (37.6) | 0.734 |
| **CRT** | | 117 (43.0) | 37 (44.0) | 78 (45.1) | 0.103 |
| **eGFR <50**** | | 83 (30.5) | 28 (33.3) | 51 (29.5) | 0.441 |
| **LVEF (%)** | | 35 [27, 43] | 32 [25, 45] | 35 [30, 43] | 0.203 |
| **NYHA** | I-II | 199 (73.2) | 56 (66.7) | 133 (76.9) | 0.187 |
|  | III-IV | 61 (22.4) | 24 (28.6) | 36 (20.8) |  |
| **QRS width (ms)** | | 114 [100, 145] | 116 [97, 150] | 114 [100, 140] | 0.730 |
| **ACEi/ARB** | | 212 (77.9) | 65 (77.4) | 135 (78.0) | 0.871 |
| **Amiodarone** | | 126 (46.3) | 39 (46.4) | 79 (45.7) | 0.638 |
| **BB** | | 229 (84.2) | 72 (85.7) | 145 (83.8) | 0.750 |
| **MRA** | | 106 (39.0) | 32 (38.1) | 69 (39.9) | 0.889 |
| **Statin** | | 88 (32.4) | 27 (32.1) | 57 (32.9) | 0.355 |

*Values are n (%) or median [IQR]. * = 15 out of 272 had missing data for time to ablation, ** = 7 patients out of 257 had missing data for eGFR*.

ACEi = ACE inhibitor, ARB = angiotensin receptor blocker, BB = beta blocker, COPD = chronic obstructive pulmonary disease, CRT = cardiac resynchronization therapy, DM = diabetes mellitus, eGFR = estimated glomerular filtration rate (ml/min/1.73m^2^), HT = hypertension, MRA = mineralocorticoid receptor antagonist, NYHA = New York Heart Association classification of heart failure

### Baseline characteristics of patients that experienced a VT during follow-up (P-DCM) or at baseline (VT-DCM):

Supplemental Table 3. Baseline characteristics of patients that experienced a VT during follow-up (P-DCM) or at baseline (VT-DCM)

|  | | **P-DCM** | **VT-DCM** | **P value** |
| --- | --- | --- | --- | --- |
| **N** | | 117 | 272 |  |
| **Age** | | 62 [57-69] | 62 [51-71] | 0.918 |
| **Female** | | 19 (16.2) | 41 (15.1) | 0.890 |
| **COPD** | | 13 (11.1) | 20 (7.4) | 0.451 |
| **DM** | | 24 (20.5) | 51 (18.8) | 0.107 |
| **HT** | | 26 (22.2) | 121 (44.5) | <0.001 |
| **Stroke** | | 11 (9.4) | 7 (2.6) | 0.001 |
| **AFib** | | 58 (49.6) | 100 (36.8) | 0.014 |
| **CRT** | | 61 (52.1) | 117 (43.0) | 0.006 |
| **eGFR <50** | | 15 (12.8) | 83 (30.5) | <0.001 |
| **LVEF (%)** | | 20 [16- 26] | 35 [27-43] | <0.001 |
| **NYHA** | I/II | 55 (47.0) | 199 (73.2) | <0.001 |
|  | III/IV | 62 (53.0) | 61 (22.4) |  |
| **QRS width (ms)** | | 138 [114-164] | 114 [100-145] | <0.001 |
| **ACEi/ARB** | | 115 (98.3) | 212 (77.9) | <0.001 |
| **Amiodarone** | | 4 ( 3.4) | 126 (46.3) | <0.001 |
| **BB** | | 98 (83.8) | 229 (84.2) | 0.792 |
| **MRA** | | 76 (65.0) | 106 (39.0) | <0.001 |
| **Statin** | | 47 (40.2) | 88 (32.4) | 0.277 |

Values are n (%) or median [IQR].

ACEi = ACE inhibitor, ARB = angiotensin receptor blocker, BB = beta blocker, COPD = chronic obstructive pulmonary disease, CRT = cardiac resynchronization therapy, DM = diabetes mellitus, eGFR = estimated glomerular filtration rate (ml/min/1.73m^2^), HT = hypertension, MRA = mineralocorticoid receptor antagonist, NYHA = New York Heart Association classification of heart failure

### Landmark analysis:

Patients that were alive at the breakpoint time and had had a VT within the preceding year (or 6 months for the first breakpoint) were compared to patients without VT. As occurrence of VT was a rare event in the DANISH cohort, the analysis was performed on the entire dataset. The landmark analysis shows that the risk of the primary endpoint is stable over time regardless of occurrence/recurrence of VT, with a HR of 3.40 (2.02 – 5.72) at 6 months and 3.14 (1.86 – 5.31) at 12 months. From 2 years and beyond, very few patients had a VT in the preceding year making the results unreliable.

Supplemental Table 4. Landmark analysis.

**6 months**

| Group | Patients (n) | Events | Unadjusted HR (95% CI) |
| --- | --- | --- | --- |
| No VT | 696 | 112 | Reference |
| VT | 61 | 17 | 3.40 (2.02 – 5.72) |

**1 year**

| Group | Patients (n) | Events | Unadjusted |
| --- | --- | --- | --- |
| No VT | 650 | 92 | Reference |
| VT | 77 | 17 | 3.14 (1.86 – 5.31) |

**2 years**

| Group | Patients (n) | Events | Unadjusted |
| --- | --- | --- | --- |
| No VT | 633 | 78 | Reference |
| VT | 8 | 1 | 1.36 (0.19 – 9.78) |

**3 years**

| Group | Patients (n) | Events | Unadjusted |
| --- | --- | --- | --- |
| No VT | 508 | 46 | Reference |
| VT | 6 | 1 | 1.83 (0.25 – 13.27) |

**4 years**

| Group | Patients (n) | Events | Unadjusted |
| --- | --- | --- | --- |
| No VT | 406 | 22 | Reference |
| VT | 0 | 0 | Not applicable |

### Early occurrence/recurrence of VT:

Early occurrence/recurrence of VT (within 31 days) appears to raise the risk of the primary endpoint further (unadjusted HR 6.34 [95% CI: 3.63 – 11.06]).

Early occurrence/recurrence of VT (within 31 days) appears to also raise the risk of the secondary endpoint further (unadjusted HR 10.89 [95% CI: 5.82 – 20.39]).

Early recurrence is likely due to a more extensive VT substrate difficult to control by ablation. The higher risk of all-cause mortality and non-sudden cardiac death in this subgroup supports the assumption that the presence of a VT substrate may indicate a critical amount of fibrosis.
